# Analysis of factors influencing anxiety in patients undergoing thoracoscopic surgery for lung tumors

**DOI:** 10.3389/fpsyg.2026.1828073

**Published:** 2026-05-11

**Authors:** Yan Li, Bingbing Li, Tianhao Xie, Chenye Shao, Huanhuan Fan, Hao Ding, Ling Yang

**Affiliations:** 1 Department of Thoracic Surgery, The First Affiliated Hospital of Soochow University, Suzhou, China; 2 Institute of Minimally Invasive Thoracic Cancer Therapy and Translational Research, Soochow University, Suzhou, China

**Keywords:** anxiety, lung tumor, risk factors, self-rating anxiety scale, thoracoscopic surgery

## Abstract

**Objective:**

This study investigated preoperative anxiety levels in patients undergoing thoracoscopic surgery for lung tumors and analyzed factors influencing postoperative changes in anxiety, aiming to provide a scientific basis for psychological intervention.

**Methods:**

We enrolled 213 patients who underwent thoracoscopic surgery for lung tumors at the First Affiliated Hospital of Soochow University between July 2024 and August 2025. Data were collected using a general information questionnaire and the Self-Rating Anxiety Scale (SAS). Univariate and multivariate logistic regression analyses identified factors associated with preoperative anxiety and postoperative changes in anxiety levels.

**Results:**

The prevalence of preoperative anxiety symptoms was 19.72%. Multivariate logistic regression identified comorbid hypertension *OR* = 5.094;*P* < 0.05 as an independent risk factor for preoperative anxiety. Postoperatively, anxiety scores decreased in 84 patients (39.44%) but increased in 129 patients (60.56%), with an overall symptom prevalence of 15.49%. Age over 58 years *OR* = 4.588, malignant pathology *OR* = 4.222, duration of surgery over 90 min *OR* = 3.789, a postoperative hospital stay exceeding 3 days *OR* = 4.632 and drainage volume exceeding 200 ml *OR* = 5.031, were significant risk factors for increased postoperative anxiety (*P* < 0.05).

**Conclusions:**

Comorbid hypertension significantly influences preoperative anxiety in these patients. Furthermore, age greater than 58 years, a malignant pathological diagnosis, and a postoperative hospitalization longer than 3 days are important factors associated with elevated postoperative anxiety.

## 1 Introduction

Lung cancer is the most common malignant tumor and the leading cause of cancer-related death globally (Lim et al., 2021). Advances in multi-slice spiral CT imaging and thoracoscopic minimally invasive surgical systems have enabled significant progress in the early screening and precise treatment of lung tumors, substantially improving patient 5-year survival rates (Huang et al., 2020). The diagnosis and treatment process, however, frequently triggers negative emotional states such as depression, anxiety, and pessimism. Perioperative anxiety represents a critical clinical concern, as it can adversely influence treatment outcomes, recovery trajectories, and long-term survival (Ferreira et al., 2021). Evidence-based research indicates that the standardized prevalence of anxiety disorders in patients with malignant tumors reaches 19.0%, with lung cancer patients demonstrating particular psychological vulnerability (Dunstan and Scott, 2020; Leung et al., 2021). This multidimensional stress response can suppress immune function via the hypothalamic-pituitary-adrenal axis—elevating PD-L1 expression and reducing CD8+ levels in the tumor microenvironment—and may reduce treatment adherence, increasing the risk of postoperative complications by 12%−15% and ultimately affecting tumor-specific survival (Smolarz et al., 2025).

Total thoracoscopic lobectomy is now widely adopted in thoracic surgery due to its high feasibility and minimal patient harm (Xu et al., 2014). The most common technique is single-port video-assisted thoracoscopic surgery, which limits damage to the chest wall muscles and promotes postoperative recovery (Park et al., 2016). Regardless of the specific approach, however, the surgery itself constitutes a potent stressor that can induce anxiety, depression, and behavioral changes. In severe instances, these psychological factors contribute to perioperative complications (Lim et al., 2022). The factors influencing anxiety in patients undergoing thoracoscopic lung tumor surgery are complex and multifactorial, encompassing disease characteristics, individual differences, and surgical intervention. To effectively alleviate patient anxiety and enhance recovery quality, we systematically investigated and analyzed these factors. Based on existing research data, this study explores the influencing factors and mechanisms of anxiety in this patient population, aiming to furnish a scientific foundation and practical guidance for subsequent clinical psychological intervention.

## 2 Methods

### 2.1 Participants

This study enrolled 213 patients who underwent thoracoscopic surgery for lung tumors at the Department of Thoracic Surgery, the First Affiliated Hospital of Soochow University, between July 2024 and August 2025. The cohort comprised 66 males (30.99%) and 147 females (69.01%), all aged 18 years or older. Data were collected using a general questionnaire and the Self-Rating Anxiety Scale (SAS). Of 237 questionnaires distributed, 24 were excluded due to incompleteness, resulting in 213 valid responses and an effective response rate of 89.9%.

### 2.2 Exclusion criteria

Exclusion criteria included the use of anti-anxiety, antidepressant, or antipsychotic medications within the 3 months prior to the survey, as well as the presence of brain metastases. All participants provided informed consent after understanding the study procedures.

### 2.3 Research methods

#### 2.3.1 General clinical data

Collected patient data included name, gender, age, marital status, body mass index (BMI), smoking history, alcohol consumption history, history of underlying diseases (e.g., hypertension and diabetes), previous surgical history, time of tumor discovery, duration of pre- and post-operative hospital stay, surgical procedure, surgery duration, surgical pathology diagnosis, type of post-operative drainage tube, drainage volume, and time of drain removal.

#### 2.3.2 Assessment tool

Anxiety was screened using the Zung SAS (Oudkerk et al., 2021). This 20-item scale employs a four-point rating system (1–4 points), yielding a total raw score between 20 and 80. The raw score is multiplied by 1.25 to obtain a standardized score ranging from 25 to 100.A standardized score below 45 is considered normal, while a score of 45 or higher indicates anxiety, with 45–54 classified as mild, 55–64 as moderate, and 65–100 as severe anxiety.

#### 2.3.3 Procedures

Researchers received training to ensure adherence to standardized survey criteria and procedures. General data were collected and entered into the hospital's internal database. For SAS data, researchers followed up with patients within 3 days before and after surgery to obtain the information. Researchers explained the survey purpose, key considerations, and completion instructions to patients. Patients completed the SAS scale independently; when this was not possible, a nurse explained each item and recorded the responses on their behalf.

#### 2.3.4 Statistical analysis

All data were analyzed using SAS 9.4 software. Non-normally distributed continuous variables were expressed as median and interquartile range, while categorical variables were expressed as percentages (frequencies); variable assignments are presented in Table 1. Univariate logistic regression was first applied to all data to identify statistically significant risk factors (*p* < 0.05). These significant factors were then entered into a multivariate logistic regression model, with *p* < 0.05 considered statistically significant.

**Table 1:** Variable assignment instructions.

Variable	Assignment explanation
Age	≤ 58 = 0; >58 = 1
Gender	male = 0; female = 1
Marital status	No = 0; Yes = 1
BMI	Normal = 0; High = 1; Low = 2
Smoking history	No = 0; Yes = 1
Alcohol consumption history	No = 0; Yes = 1
History of hypertension	No = 0; Yes = 1
History of diabetes	No = 0; Yes = 1
Other medical history	No = 0; Yes = 1
Time of tumor discovery	≤ 3 months = 0; >3 months = 1
Previous surgical history	No = 0; Yes = 1
Histopathology	Benign = 0; Malignant = 1
Surgical method	Lobectomy = 0; Sublobectomy = 1
Duration of surgery	≤ 90 min = 0; >90 min = 1
Preoperative hospital stay	≤ 3 days = 0; >3 days = 1
Postoperative hospital stay	≤ 3 days = 0; >3 days = 1
Type of drainage tube	Thoracic drainage tube = 0; Silicone drainage tube = 1
Drainage volume	< 200 = 0; ≥200 = 1
Postoperative extubation time	≤ 2 days = 0; >2 days = 1
Preoperative anxiety state	Normal = 0; Mild anxiety = 1; Moderate anxiety = 2;
Changes in anxiety conditions	decrease = 0; increase = 1

## 3 Results

### 3.1 Baseline characteristics

This retrospective study included 213 patients who underwent thoracoscopic surgery for lung tumors at the First Affiliated Hospital of Soochow University between July 2024 and August 2025. The cohort comprised 66 male (30.99%) and 147 female (69.01%) patients. Patient age ranged from 23 to 82 years, with a mean of 55.17 ± 13.70 years, and the cohort was stratified according to the median age of 58 years. These demographic characteristics are presented in Table 2.

**Table 2:** Patient baseline information.

Patient's clinical data	Category	Result
Age [Years (25%, 75%)]		58 (46.66)
Gender [Cases (%)]	Male	66 (30.99)
	Female	147 (69.01)
Marital status [Cases (%)]	Yes	207 (97.18%)
	No	6 (2.82%)
BMI [Cases (%)]	Normal	99 (46.48%)
	High	15 (7.04%)
	Low	99 (46.48%)
Smoking history [Cases (%)]	Yes	30 (14.08%)
	No	183 (85.92%)
Alcohol consumption history [Cases (%)]	Yes	27 (12.68%)
	No	186 (87.32%)
History of underlying diseases [Cases (%)]	History of hypertension [Cases (%)]	57 (26.76)
	History of diabetes [Cases (%)]	6 (2.82)
	Other medical history [Cases (%)]	18 (8.45)
Time since diagnosis [months (25%, 75%)]		24 (1.24)
Previous surgical history [Cases (%)]		93 (43.66)
Histopathology [Cases (%)]	Malignant	168 (78.87)
	Benign	45 (21.13)
Surgical method [Cases (%)]	Lobectomy	42 (19.72)
	Sublobectomy	171 (80.28)
Duration of surgery [minutes (25%, 75%)]		90 (65.120)
Preoperative hospital stay [Cases (%)]	≤ 3 days	102 (47.89)
	>3 days	111 (52.11)
Postoperative hospital stay [Cases (%)]	≤ 3 days	138 (64.79)
	>3 days	75 (35.21)
Type of drainage tube [Cases (%)]	Thoracic drainage tube	184 (64.89)
	Silicone drainage tube	75 (35.21)
Postoperative extubation time [Cases (%)]	≤ 2 days	132 (61.97)
	>2 days	81 (38.03)
Drainage volume [ml (%)]	< 200	171 (80.28)
	≥200	42 (19.72)

### 3.2 Preoperative anxiety status and univariate analysis in patients undergoing thoracoscopic surgery for lung tumors

Among the 213 patients undergoing thoracoscopic surgery for lung tumors, 42 (19.72%) reported experiencing anxiety within the 3 days prior to surgery; this comprised 36 cases of mild anxiety (85.71%) and 6 cases of moderate anxiety (14.29%). Univariate analysis examined preoperative patient characteristics, including age, gender, marital status, BMI, smoking history, alcohol use, underlying conditions such as hypertension and diabetes, time since tumor discovery, and previous surgical history. The analysis revealed that preoperative anxiety was significantly associated with a history of hypertension and the time of tumor discovery (*P* < 0.05), as detailed in Table 3.

**Table 3:** Univariate logistic regression analysis of patients' preoperative anxiety levels.

Variable	Category	Cases (%)	Correction OR (95% CI)	*P* value	*X* ^2^
Age	≤ 58 years old	114 (53.53)	Control group		
	>58 years old	99 (46.48)	0.801 (0.229–2.802)	0.7280	0.1209
Gender	Male	66 (30.99)	Control group		
	Female	147 (69.01)	5.699 (0.712–45.612)	0.1010	2.6889
Marital status [Cases (%)]	Yes	207 (97.18)	Control group		
	No	6 (2.82)	0.067 (0.004–1.047)	0.0539	3.7143
BMI [Cases (%)]	Normal	99 (46.48)	Control group		
	High	15 (7.04)	1.333 (0.120–14.798)	0.8147	0.0549
	Low	99 (46.48)	1.236 (0.339–4.511)	0.7486	0.1027
Smoking history	No	183 (85.92)	Control group		
	Yes	30 (14.08)	1.214 (0.221–6.671)	0.8234	0.0498
Alcohol consumption history	No	186 (87.32)	Control group		
	Yes	27 (12.68)	0.567 (0.063–5.070)	0.6114	0.2581
History of hypertension	No	156 (73.24)	Control group		
	Yes	57 (26.76)	5.313 (1.440–19.606)	0.0122	6.2859
History of diabetes	No	207 (97.18)	Control group		
	Yes	6 (2.82)	< 0.001	0.9796	0.0007
Other medical history	No	195 (91.55)	Control group		
	Yes	18 (8.45)	3.552 (0.392–32.154)	0.9623	1.2719
Time since diagnosis	>3 months	78 (36.62)	Control group		
	≤ 3 months	135 (63.38)	4.075 (1.093–15.188)	0.0363	4.3810
Previous surgical history	No	120 (56.34)	Control group		
	Yes	93 (43.66)	0.358 (0.088–1.460)	0.1521	2.0514

### 3.3 Multivariate logistic regression model analysis of preoperative anxiety in patients undergoing thoracoscopic surgery for lung tumors

A multivariate logistic regression model was constructed to analyze preoperative anxiety levels in patients undergoing thoracoscopic surgery for lung tumors. The dependent variable was preoperative anxiety, and the independent variables were the factors significantly associated with anxiety in the univariate analysis. A history of hypertension emerged as a statistically significant predictor. This finding indicates that a history of hypertension increases the risk of elevated preoperative anxiety in these patients, with an odds ratio of 5.094 (*P* < 0.05; Table 4).

**Table 4:** Multivariate logistic regression model analysis of the preoperative anxiety level of the patients.

Variable	Correction OR (95% CI)	Regression coefficient	Standard error	*P* value	*X* ^2^
History of hypertension	5.094 (1.302–19.931)	1.6280	0.6961	0.0193	5.4696
Time since diagnosis	3.893 (0.983–15.409)	1.3591	0.7019	0.0528	3.7491

### 3.4 Changes in anxiety levels among lung cancer patients and univariate analysis

Postoperatively, anxiety was present in 15.49% of patients (33 patients: 30 mild, 3 moderate). A total of 129 patients (60.56%) exhibited an increase in SAS scores from baseline, whereas scores decreased in 84 patients (39.44%). Univariate analysis was conducted for age, gender, medical history (including hypertension and diabetes), time of tumor discovery, prior surgical history, pathological findings, surgical method, operative duration, duration of pre- and post-operative hospital stay, type of post-operative drain, drainage volume, and time of drain removal. This analysis revealed that changes in patient anxiety levels showed statistically significant associations with age, pathological findings, operative duration, post-operative hospital stay duration, and drainage volume (*P* < 0.05), as detailed in Table 5.

**Table 5:** Univariate logistic regression analysis of changes in patient anxiety.

Variable	Category	Cases (%)	Correction OR (95% CI)	*P* value	*X* ^2^
Age	≤ 58 years old	114 (53.53)	Control group		
	>58 years old	99 (46.48)	4.588 (1.603–13.130)	0.0045	8.0658
Gender	Male	66 (30.99)	Control group		
	Female	147 (69.01)	0.622 (0.215–1.797)	0.3807	0.7686
History of hypertension	No	156 (73.24)	Control group		
	Yes	57 (26.76)	2.221 (0.697–7.073)	0.1771	1.8218
History of diabetes	No	207 (97.18)	Control group		
	Yes	6 (2.82)	>999.999	0.9809	0.0006
Other medical history	No	195 (91.55)	Control group		
	Yes	18 (8.45)	3.552 (0.392–32.154)	0.2594	1.2719
Time since diagnosis	≤ 3 months	78 (36.62)	Control group		
	>3 months	135 (63.38)	1.208 (0.451–3.232)	0.7069	0.1414
Previous surgical history	No	120 (56.34)	Control group		
	Yes	93 (43.66)	1.718 (0.647–4.566)	0.2777	1.1782
Histopathology	Benign	45 (21.13)	Control group		
	Malignant	168 (78.87)	4.222 (1.257–14.174)	0.0198	5.4323
Surgical method [Cases (%)]	Lobectomy	42 (19.72)	Control group		
	Sublobectomy	171 (80.28)	0.550 (0.154–1.965)	0.3576	0.8464
Duration of surgery [minutes (25%, 75%)]	≤ 90 min	120 (56.34)	Control group		
	>90 min	93 (43.66)	3.789 (1.331–10.785)	0.0125	6.2324
Preoperative hospital stay [Days (25%, 75%)]	≤ 3Days	102 (47.89)	Control group		
	>3Days	111 (52.11)	1.852 (0.707–4.851)	0.2098	1.5728
Postoperative hospital stay [Days (25%, 75%)]	≤ 3Days	138 (64.89)	Control group		
	>3Days	75 (35.21)	4.000 (1.282–12.478)	0.0169	5.7034
Type of drainage tube [Cases (%)]	Thoracic drainage tube	138 (64.89)	Control group		
	Silicone drainage tube	75 (35.21)	0.964 (0.356–2.609)	0.9429	0.0051
Postoperative extubation time [Days (25%, 75%)]	≤ 3Days	132 (61.97)	Control group		
	>3Days	81 (38.03)	2.609 (0.918–7.413)	0.0720	3.2378
Drainage volume [ml (%)]	< 200	171 (80.28)	Control group		
	≥200	42 (19.72)	5.031 (1.031–24.545)	0.0457	3.9913

### 3.5 Multivariate logistic regression model analysis of changes in anxiety levels among patients undergoing thoracoscopic surgery for lung tumors.

Changes in anxiety levels among patients undergoing thoracoscopic surgery for lung tumors served as the dependent variable, while factors significantly associated with anxiety changes in the univariate analysis were included as independent variables in a multivariate logistic regression model. The analysis identified age, histopathology, and postoperative hospital stay duration as statistically significant predictors. Specifically, age over 58 years, a malignant pathological diagnosis, and a postoperative hospital stay exceeding 3 days were associated with an increased risk of elevated anxiety. The odds ratios were 4.550 for age, 4.722 for histopathology, and 4.632 for postoperative hospital stay duration, with all *P* values < 0.05. These results are presented in Table 6.

**Table 6:** Multivariate logistic regression analysis of changes in patient anxiety.

Variable	Correction OR (95% CI)	Regression coefficient	Standard error	*P* value	*X* ^2^
Age	4.355 (1.255–15.110)	1.4713	0.6347	0.0205	5.3725
Histopathology	4.681 (1.078–20.323)	1.5435	0.7491	0.0394	4.2457
Duration of surgery	2.545 (0.747–8.667)	0.9342	0.6252	0.1351	2.2330
Postoperative hospital stay	4.357 (1.089–17.423)	1.4717	0.7072	0.0374	4.3309
Drainage volume	1.293 (0.206–8.103)	−2.2980	0.8146	0.0048	7.9579

## 4 Discussion

This study investigated patients undergoing thoracoscopic surgery for lung tumors, revealing a preoperative anxiety prevalence of 19.72%, which is consistent with rates reported for other cancer populations. While the absolute number of patients meeting the clinical threshold for anxiety decreased slightly postoperatively, a significant proportion (60.56%) exhibited increased anxiety scores relative to their preoperative baseline. This pattern indicates a risk for heightened anxiety following a lung tumor diagnosis and a trend toward increased anxiety after surgery, aligning with a survey of thoracic surgery cancer patients that also found greater severity of postoperative anxiety (Deng and Chen, 2023). Furthermore, lung cancer patients frequently experience postoperative pain and poor sleep quality (Wang et al., 2020), and some face complications such as pulmonary infection. These adverse reactions can negatively impact both physical and psychological wellbeing, thereby impeding recovery. Consequently, clinicians should provide thorough preoperative explanations and psychological support, maintain postoperative communication, and deliver attentive nursing care to mitigate patient anxiety and distress.

We identified comorbid hypertension as a significant predictor of preoperative anxiety, a finding consistent with meta-analyses demonstrating a strong correlation between anxiety and hypertension (Li et al., 2022). Chronic conditions like hypertension cause long-term cardiovascular strain, imposing considerable physical and mental stress. The combined burden of comorbidity and malignancy likely lowers the threshold for negative emotional responses. Research further indicates that coexisting anxiety and hypertension significantly compromise the quality of life in cancer patients (Linden et al., 2012). Therefore, when treating patients with hypertension, healthcare professionals should closely monitor the emotional state of those with chronic diseases, enhance patient support and psychological counseling, and work to alleviate their anxieties.

Regarding postoperative anxiety trajectories, age over 58 years emerged as a significant risk factor. Patients in this age group often retain substantial family responsibilities and social roles, which can lead to complex psychological changes during treatment. Furthermore, older patients may experience heightened fear and anxiety due to an incomplete understanding of their disease. In clinical practice, the complex physiological and psychosocial circumstances of elderly patients are frequently overlooked amid concerns about age and comorbidities. Although advanced age is sometimes considered protective against anxiety, studies show that anxiety prevalence is higher among elderly cancer patients than in the general elderly population (Cui et al., 2021). Clinicians should therefore recognize the complex emotional challenges older patients may face and increase clinician-patient communication and supportive care to help them approach treatment with a more positive attitude.

Prolonged hospitalization (>3 days) was also associated with increased anxiety. As treatment continues, extended hospital stays can exacerbate patient anxiety (Zhang et al., 2025). Prolonged hospitalization may disrupt sleep, and the constant clinical environment can make patients feel a loss of personal control and security. Therefore, admitting departments should optimize preoperative pathways, enhance protocols for postoperative recovery, and strive to minimize unnecessary hospitalization.

Furthermore, surgery time, hospital stay, and postoperative drainage volume are usually simple and direct indicators for patients to self-assess the severity of their condition, thus directly impacting their anxiety levels. Therefore, doctors continuously learning more refined surgical techniques and employing more advanced surgical instruments and more suitable biomaterials can not only reduce complications but also significantly reduce patient anxiety.

In summary, despite growing public awareness of tumors, a cancer diagnosis inevitably triggers emotional fluctuations, often dominated by negative emotions. A thorough understanding of anxiety levels in lung cancer patients and their changes across the perioperative period is crucial for implementing optimal clinical management and selecting the most effective nursing interventions.

### 4.1 Study limitations

This study has several limitations. First, the sample was drawn from a single hospital and was relatively small *N* = 213. Second, the follow-up period was short. Future multi-center studies with larger samples and longer follow-up are required to better elucidate the factors influencing anxiety in lung cancer patients and the long-term psychological effects of surgical intervention.

## 5 Conclusions

In conclusion, patients undergoing thoracoscopic surgery for lung tumors frequently experience perioperative anxiety, a condition that often intensifies after the operation. Preoperative anxiety shows a positive correlation with both hypertension and the duration since tumor discovery. Advanced age (>58 years), a malignant pathological diagnosis, and a prolonged hospital stay are identified as risk factors for heightened postoperative anxiety. Consequently, implementing appropriate perioperative psychological assessments for these patients is warranted. Enhanced doctor-patient communication and personalized psychological interventions for high-risk groups are necessary to mitigate these negative psychological impacts.

## Data Availability

The raw data supporting the conclusions of this article will be made available by the authors, without undue reservation.
